# Screening of mushrooms from the woodlands of Zimbabwe: Occurrence of lectins and partial purification of a mucin specific lectin from *Boletus edulis*

**DOI:** 10.1371/journal.pone.0265494

**Published:** 2022-04-14

**Authors:** Tsungai Reid, Faith Fore, Farisai Chidzwondo, Chenjerayi Kashangura, Babill Stray-Pedersen, Takafira Mduluza

**Affiliations:** 1 Biochemistry Department, University of Zimbabwe, Harare, Zimbabwe; 2 Kutsaga Research Station, Harare, Zimbabwe; 3 Institute of Clinical Medicine, University in Oslo, Oslo University Hospital, Oslo, Norway; 4 School of Laboratory Medicine and Medical Sciences, University of KwaZulu Natal, Durban, South Africa; MGR College, INDIA

## Abstract

Mushrooms are known to possess a diversity of bioactive compounds that include lectins, which are proteins or glycoproteins that bind specifically to cell surface carbohydrates, culminating in cell agglutination. The present study describes the screening of lectin activity from ten local mushrooms, namely, *Amanita zambiana*, *Boletus edulis*, *Cantharellus heinemannianus*, *Cantharellus miomboensis*, *Cantharellus symoensii*, *Lactarius kabansus*, *Amanita* sp., *Coprinus* sp., *Ganoderma lucidum* and *Trametes strumosa*. The lectin content was detected by the haemagglutination activity of mushrooms against sheep and goat erythrocytes. Among the different mushrooms screened *Amanita* sp., *Boletus edulis* and *Lactarius kabansus* showed high lectin activity (39, 617 and 77 HAU/mg mushroom, respectively). *Boletus edulis* was used for the haemagglutination inhibition assay. A total of twenty sugars and sugar derivatives, namely, α-lactose, D-glucose, D-mannose, D-raffinose, N-acetyl glucosamine, maltose, melibiose, D-ribose, porcine mucin, D-cellobiose, D-arabinose, α-methyl-D-glucoside, methyl-α-D-mannopyranoside, D-trehalose, L-arabinose, L-sorbose, L-lyxose, β-lactose, DL-xylose, and D-galactose, were used for the haemagglutination inhibition assay. Of the various carbohydrates tested, only porcine mucin was found to be the most potent inhibitor of *Boletus* lectin. The lectin from *Boletus* mushroom was partially purified using ammonium sulphate precipitation. The highest lectin activity was observed in the 30%-60% fraction. This study revealed for the first time the occurrence of lectins in the local Zimbabwean mushrooms studied as well as isolation of a novel mucin-specific lectin. The information obtained can be used for further investigation of cell surface sugars, purification and characterisation of glycoproteins and their contribution towards the medicinal properties of local mushrooms.

## Introduction

Lectins are proteins or glycoproteins of non-immune origin which can bind specifically and reversibly to complex carbohydrates that are abundant on cell surfaces, resulting in agglutination of cells or precipitation of glycoconjugates [1–5]. These molecules are widely distributed in nature and found in all kinds of organisms such as plants, fungi, including mushrooms, animals, bacteria, and viruses [6]. There is an increased research interest in lectins isolated from fungi, in particular, mushrooms. Up to date, over 100 different mushroom lectins have been identified [7].

Mushroom lectins have drawn the attention of many researchers, mainly due to their exploitable properties that encompass a wide range of biological activities such as antimicrobial, antiproliferative, antitumor, anti-insect, hypotensive, immunomodulatory, mitogenic and anti-HIV-1 reverse transcriptase activities [8–13]. *Agaricus bisporus* lectin has antiproliferative action against human colon cancer and breast cell lines [14], while the *Pholiota adiposa* lectin has HIV inhibitory activity by targeting the reverse transcriptase and also antiproliferative activity towards hepatoma Hep G2 cells [15]. *Volvariella volvacea* lectin possesses antitumor activity to sarcoma S-180 cells while *Boletus* lectins have mitogenic activity towards tumour cells [16–18] and antimicrobial activity [19].

The detection of lectins relies on their ability to agglutinate red blood cells and lectins inhibition by a specific sugar, which is a major attribute of these proteins [1, 16, 17, 20–22]. Accumulation of lectins in crude extracts of mushrooms is detected by haemagglutination assay using human (A, B and O blood groups) and animal (goose, goat, rabbit, rat and sheep erythrocytes [23]. The carbohydrate binding properties of lectins are critically important not only in clarifying their biological roles, but in developing them as carbohydrate probes or medicines. For example, the HIV surface glycoproteins are used as targets for action of lectin as antiviral agents [24]. Carbohydrate binding properties of lectins are applied in the fields of immunology, cell biology, cancer research and genetic engineering. The *α*1-2-fucose-specific lectin (rBC2LCN) is used in the detection of undifferentiated induced pluripotent stem cells, but not differentiated somatic cells. The lectin from *Maackia amurensis* is used in prostate cancer diagnosis due to its preferential binding to prostate-specific antigen [25]. In addition, the newly developed lectin array is increasingly being used as a powerful tool for the comprehensive analysis of glycans or glycoconjugates. Mushroom lectins are considered potent therapeutic agents [1, 10, 26].

High content of lectins in mushrooms has been detected in diverse species of genera *Lactarius*, *Russula*, *Boletus*, *Phallus*, *Amanita* and *Hygrophorus* [27, 28]. Mushroom lectins are highly affected by the environment, such as time of harvest, geographic location, and part of mushroom from which the lectin was isolated. The same mushroom species can have different types of lectins depending on the environment where the mushrooms were collected [20, 22]. Some mushroom lectins have been reported to be sensitive to inhibition of agglutination by more than a single sugar and/or sugar derivative, for example, lectins from *Boletus edulis* (melibiose- and xylose-specific), *Ganoderma capense* (D+-galactose and D+-galactosamine), *Ganoderma lucidum* (glucosamine, galactosamine and glycoprotein fetuin) and *Lentinus edodes* (*N*-acetylgalactosamine and *N*-acetylglucosamine-specific). Edible mushroom lectins such as lectins from *Volvariella volvacea* have specificity towards complex carbohydrates rather than simple sugars while other mushrooms such as *Agaricus edulis* have haemagglutinating activities that are not affected by simple sugars, sugar derivatives or complex glycoproteins [13].

There is less information on the occurrence of lectins from Zimbabwe mushrooms and the possible clinical significance of these mushroom lectins. Thus, Zimbabwe mushrooms represent an enormous unexplored source of potentially useful and novel lectins. Hence, in this study we aimed at screening for the occurrence of lectins from ten different mushrooms found in Zimbabwe, and we report for the first time, a novel lectin isolated from *Boletus edulis* mushroom from Miombo woodlands of Zimbabwe.

## Materials and methods

### Collection, identification and preparation of mushrooms

A total of ten different mushrooms, namely, *Amanita zambiana*, *Boletus edulis*, *Cantharellus heinemannianus*, *Cantharellus miomboensis*, *Cantharellus symoensii*, *Lactarius kabansus* (edible mushrooms), *Amanita* sp., *Coprinus* sp., *Ganoderma lucidum* and *Trametes strumosa* (non-edible mushrooms), were collected from the Miombo woodlands of Zimbabwe. Identification of the mushrooms was done by experienced plant biologists based on morphological characteristics, including colour of the mushroom cap and spore print. Final identification was done by comparing the visual appearance and the recorded characters of mushroom species with standard mushroom collection guides [27–30]. The fresh mushrooms were sliced into thin strips and sun dried for seven days. The dried mushrooms were then ground to powder using an electrical grinder (Siebtechnik steel pulverizer 2, GmbH).

### Extraction of crude mushroom protein

Total protein was extracted from the mushrooms as previously done [31]. In short, 0.5 g of mushroom powder was mixed with freshly prepared 0.15 M NaCl in the ratio 1:10 w/v. The mixture was incubated at 4°C for 48 hours. After incubation, the mixture was centrifuged at 5 200 rpm for 35 minutes using a Japson (Centrifuge Machine Digital) Centrifuge. The supernatant was collected and used in haemagglutination assay.

### Preparation of erythrocytes

Sheep and goat blood were purchased from the Animal Science Department of the University of Zimbabwe. Five millilitres of either sheep or goat blood was mixed with 0.7 mL of Alsever solution (S1 Table) in a 10 mL Falcon tube. A volume of 2 mL of blood was resuspended in 28 mL of 0.9% saline solution (S1 Table) followed by centrifugation at 3500 rpm for 15 minutes at 4°C. The supernatant was discarded, and 0.9% saline solution was added to the 30 ml mark. The washing process was repeated twice, and the resultant pellet was resuspended in 20 mL of 0.9% saline solution to give 5% erythrocytes suspension.

### Haemagglutination assay

Lectins in the crude mushroom extracts were detected by their haemagglutination activity against sheep and goat erythrocytes. The haemagglutination assay was carried out in 96-well, U-bottom Sarstedt microtiter plates according to [31, 32]. A serial two-fold dilution of the mushroom extract in microtiter (25 μL) was mixed with 50 μL of 5% sheep or goat erythrocytes suspended in 0.9% saline solution followed by incubation at room temperature for 1 hour. The first two rows containing 0.9% saline solution and erythrocytes in a plate were the negative control. The results were recorded after one hour, when the erythrocytes in the negative control had fully sedimented. The haemagglutination titre, defined as the reciprocal of the highest dilution exhibiting agglutination, represented one haemagglutination unit. Specific activity was determined as the number of haemagglutination units/mg mushroom. The assays were carried out in duplicate.

### Haemagglutination inhibition assay

*Boletus edulis* was used for the haemagglutination inhibition tests by various carbohydrates, in a manner analogous to the haemagglutination test. The sugars and sugar derivatives used were α-lactose, D-glucose, D-mannose, D-raffinose, N-acetyl glucosamine, maltose, melibiose, D-ribose, porcine mucin, D-cellobiose, D-arabinose, α-methyl-D-glucoside, methyl-α-D-mannopyranoside, D-trehalose, L-arabinose, L-sorbose, L-lyxose, β-lactose, DL-xylose and D-galactose. The inhibition assay was carried out as previously done by [15, 31–33] with minor modifications. Briefly, 25 μL of saline solution was added to all the wells of a 96 well plate except for the row which was serving as positive control and the first column which was for 100% of 0.2 M sugar solution. The sugars were serially diluted with a two-fold increment until the end of the row, discarding 25 μL at the end. An equal volume of crude extract was added to each well except for the row which was serving as the negative control. The positive control was made up of crude extract and the negative control was made up of saline solution. The plate was incubated for 30 minutes at room temperature. After 30 minutes of incubation, 50 μL of 5% red blood cells was added into each well. The plate was incubated for 2 hours at room temperature.

### Partial purification of *Boletus edulis* lectin

Partial purification of the crude lectin extract from *Boletus edulis* was carried out by ammonium sulphate precipitation and dialysis [31, 32]. Ammonium sulphate was added to *B*. *edulis* crude lectin extract to 0–30% saturation with constant stirring (S2 Table). The mixture was centrifuged at 10 000 rpm for 10 minutes. The precipitate was re-dissolved in 0.9% saline solution and the supernatant was subjected to a range of 30–60% ammonium sulphate saturation. The procedure was repeated until a pellet of 60–90% ammonium sulphate was obtained. The fractions were dialysed against two changes of 500 ml 0.9% saline solution for 4 hours. The fractions were then transferred to 1,000 mL of 0.9% saline solution and dialysed overnight at 4 ºC. Haemagglutinating activity was examined in the dialysed samples.

## Results

### Haemagglutination assay

The results of the haemagglutination assays of the ten different mushrooms in this study using sheep and goat erythrocytes, are shown in Table 1. A 96 well plate showing representative results for haemagglutination assays of three of the ten mushroom species with sheep erythrocytes is presented in the S1 Fig. The results in Table 1 show that *Amanita* species, *B*. *edulis* and *L*. *kabansus* were able to agglutinate both sheep and goat erythrocytes.

**Table 1:** Haemagglutination assay of the ten mushroom species with sheep and goat erythrocytes.

Mushroom type	Sheep erythrocytes	Goat erythrocytes
*Amanita zambiana*	-	-
*Amanita* sp.	+	+
*Boletus edulis*	+	+
*Cantharellus miomboensis*	-	-
*Cantharellus heinemannianus*	-	-
*Cantharellus symoensii*	-	-
*Coprinus* sp.	-	-
*Ganoderma lucidum*	-	-
*Lactarius kabansus*	+	+
*Trametes strumosa*	-	-

(+) denotes haemagglutination activity, (-) no haemagglutination activity observed. Three mushrooms, namely, *Amanita* sp., *Boletus edulis* and *Lactarius kabansus*, showed haemagglutination activity.

The specific haemagglutination activity of the three mushrooms varied, with *B*. *edulis* having the highest specific activity of 617 and 154 haemagglutination units (HAU)/mg mushroom in both sheep and goat erythrocytes, respectively, and *L*. *kabansus* having the lowest activity of 5 HAU/mg mushroom as shown in Table 2. However, although the three lectins from mushroom could agglutinate both sheep and goat erythrocytes, *Amanita* sp. and *L*. *kabansus* showed a little higher activity with sheep erythrocytes, while *B*. *edulis* had much higher activity with goat erythrocytes.

**Table 2:** Specific activity of crude extracts of mushrooms showing haemagglutination activity.

Mushroom type	Specific activity (HAU/ mg mushroom)	
	Sheep RBCs	Goat RBCs
***Amanita* sp**.	39	10
***Boletus edulis***	154	617
***Lactarius kabansus***	77	5

The haemagglutination titre, defined as the reciprocal of the highest dilution exhibiting agglutination, represented one hemagglutination unit (HAU). Specific activity was determined as the number of hemagglutination units/mg mushroom. *Boletus edulis* had the highest specific activity, showing high lectin content.

### Haemagglutination inhibition assay

**Fig 1:**
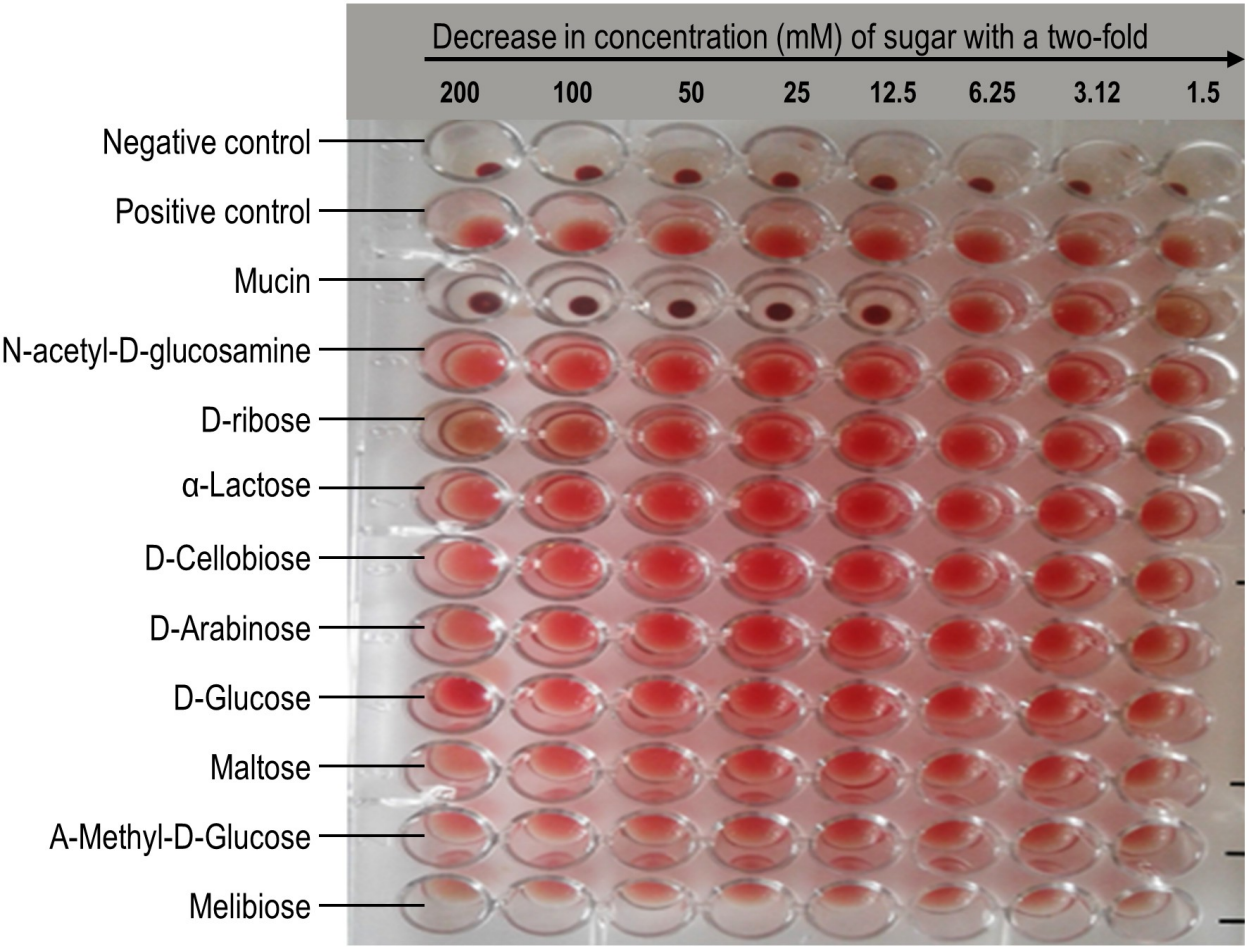
*Boletus edulis* lectin haemagglutination inhibition assay by various sugars and sugar derivatives using sheep erythrocytes. The sugars had their concentration of 0.2 M serially diluted with 0.9% saline solution from the first well to the last well of the row. Porcine mucin inhibited haemagglutination activity of crude *B*. *edulis* lectin up to 12.5 mM.

*Boletus edulis* was selected for haemagglutination inhibition assay using sheep erythrocytes. Lack of haemagglutination is the indication of specific binding and inhibitory property of the sugar (or a glycoprotein). The lowest concentration of the sugar/glycoprotein, which inhibited the agglutination, was taken as the minimal inhibitory concentration (MIC). Out of all the twenty sugars and sugar derivatives tested, only one glycoprotein, porcine mucin, was found to inhibit the haemagglutination activity of *B*. *edulis*. Porcine mucin inhibited the haemagglutination activity of the crude *B*. *edulis* lectin up to a maximum inhibitory concentration of 12.5 mM as shown in Fig 1.

**Fig 2:**
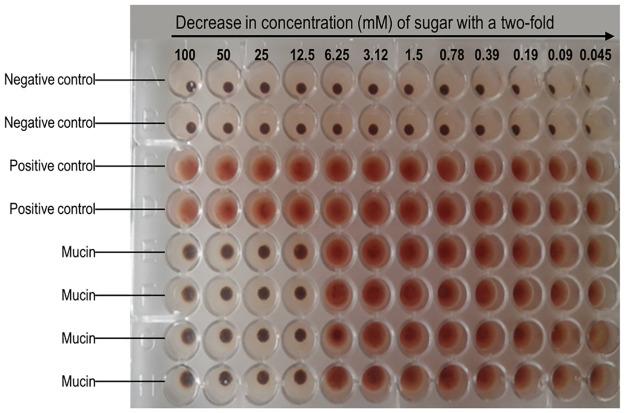
Haemagglutination inhibition assay of *B*. *edulis* lectin by porcine mucin. The maximum inhibitory concentration of porcine mucin was 12.5 mM.

Haemagglutination inhibition assay was carried out with porcine mucin to see if the results were reproducible. The same results were obtained, where porcine mucin inhibited the haemagglutination activity of crude *B*. *edulis* lectin up to a concentration of 12.5 mM as shown in Fig 2.

### Partial purification of *Boletus edulis* lectin

**Fig 3:**
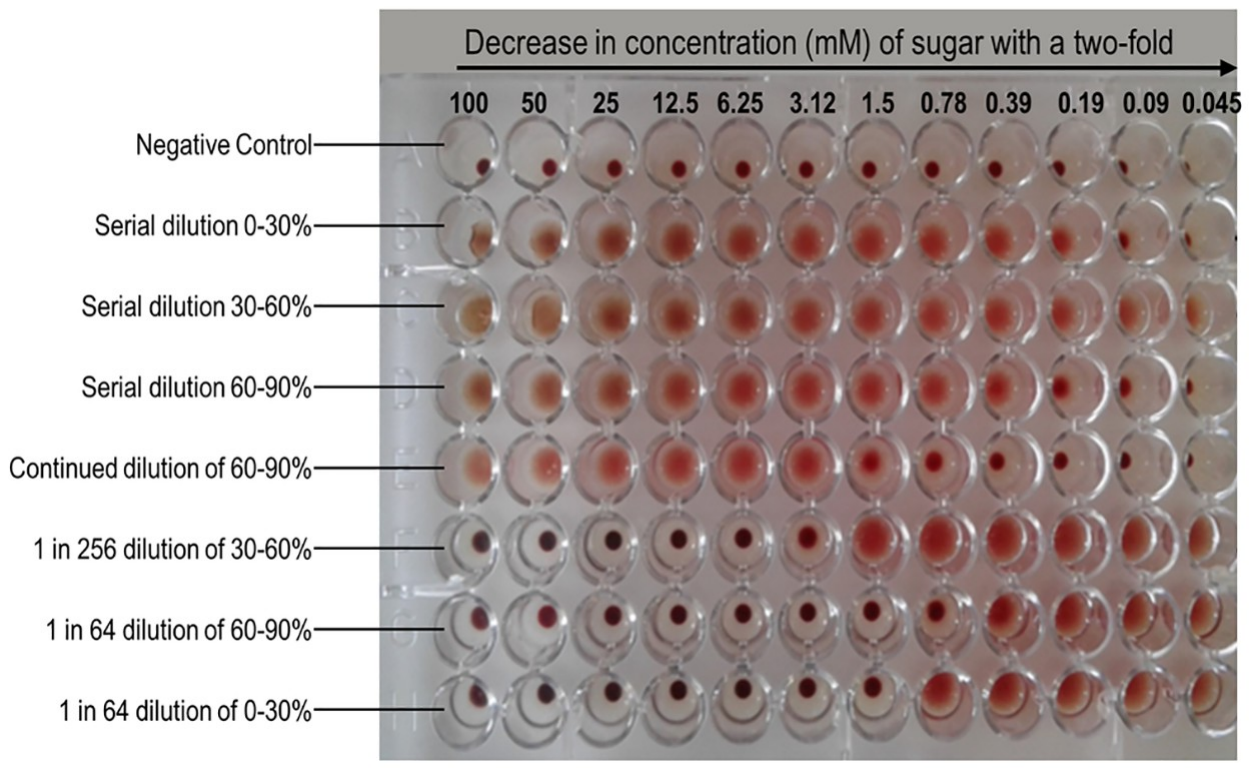
Haemagglutination and haemagglutination inhibition assay of the three partially purified fractions of *B*. *edulis*.

When the crude extract of *B*. *edulis* was subjected to different percentages of ammonium sulphate, namely, 0–30%, 30–60% and 60–90%, precipitation was observed in all the fractions. All the fractions were found to have lectin activity, however the 30–60% fraction had the highest haemagglutination activity as shown in Fig 3.

The 30–60% fraction, when serially diluted in one row, had haemagglutination activity in all the wells. Dilution was continued using the fifth row, with the fraction showing haemagglutination activity until the lectin was serially diluted 2^18^ times with 0.9% saline solution. All the fractions had their haemagglutination activity inhibited by porcine mucin up to 0.0015 M as shown in Fig 3 and Table 3.

**Table 3:** Concentration of mucin that was found to inhibit the haemagglutination of erythrocytes by the 3 fractions of *B*. *edulis* obtained from ammonium sulphate purification.

mM	100	50	25	12.5	6.25	3.12	1.78	1.56	0.78	0.39	0.19	0.09
0–30%	-	-	-	-	-	-	-	-	+	+	+	+
30–60%	-	-	-	-	-	-	+	+	+	+	+	+
60–90%	-	-	-	-	-	-	-	+	+	+	+	+

The (+) sign represents haemagglutination and the (-) sign represents no haemagglutination result. Maximum inhibitory concentration for the 30–60% fraction was 3.125 mM.

## Discussion

Mushrooms produce many kinds of proteins with important biological activities, including lectins. In this study, the lectin activity of the ten different wild mushrooms, using sheep and goat erythrocytes, were studied. The results show that *Amanita* sp., *B*. *edulis* and *L*. *kabansus* contain varying levels of lectin content. *Boletus edulis* has the highest specific activity of 617 and 154 HAU/mg mushroom in both goat and sheep erythrocytes, respectively and *L*. *kabansus* has the lowest activity of 5 HAU/mg mushroom. Haemagglutination of erythrocytes is a result of crosslinks which are formed between erythrocytes’ glyco-conjugates and the lectin. The crosslinks will form a suspension which will prevent the cells from settling at the bottom of the well [34]. In other related studies, high content of lectins in mushrooms has been detected in *B*. *edulis*, *Lactarius* and *Amanita* sp. [20, 35]. Lectins obtained from mushrooms are reportedly able to bind to abnormal and cancer cells and label these cells for destruction by the human body’s immune system [36] using a mechanism reminiscence to the complement system. Thus, the mushrooms under study, particularly *B*. *edulis* and *L*. *kabansus* could potentially be an excellent source of cancer-fighting macromolecules and can be used directly in the human diet to promote health. Extracts from the mushrooms can be obtained and commercialised as dietary supplements for their ability to enhance immune function and antitumour activity.

Lectins are classified according to the type of sugar that specifically inhibit its haemagglutination activity. Some lectins are inhibited by simple sugars such as glucose and galactose while other complex lectins are not. In another study carried out [17], *Boletus* species was tested for its sugar specificity but none of the tested sugars were able to inhibit the haemagglutination activity. Complex lectins are inhibited by glycoproteins and these glycoproteins are either O-linked or N-linked. In this study, only porcine mucin was able to inhibit the haemagglutination activity of *B*. *edulis* lectin indicating that the lectin is specific to the glycoprotein. When porcine mucin and *B*. *edulis* were incubated, the binding sites of the lectin were able to bind to porcine mucin, as a result no site was left to interact with carbohydrate moieties on erythrocytes [34]. This resulted in inhibition of haemagglutination as shown in Fig 3. The results obtained from this study indicate that the binding sites of *B*. *edulis* lectin are complex since all the simple sugars tested were not able to inhibit the haemagglutination activity of the lectin. This observation is in general agreement with previous studies where mushroom lectins which were found to be inhibited by porcine mucin could not be inhibited by simple sugars [31, 37–42].

Studies have shown that lectins isolated from mushrooms differ depending on geographical locations. The same mushroom species can have different types of lectins depending on where the mushrooms were collected. *Grifola frondosa*, for example, has been studied by two different groups of scientists and in each case, it was inhibited by a different carbohydrate, namely, N-acetyl-D-galactosamine [33] and porcine mucin [39]. Other *Boletus edulis* lectins which have been isolated are D-galactose [16] and D-lactose specific [43]. Thus, there is a huge possibility that the lectins isolated from this study are different from the ones isolated from other geographical locations. Other factors that affect the type of lectin, besides geographical origin, are the part where the lectin was isolated from the mushroom. Two lectins were isolated from *Grifola frondosa*, a terminal N-acetyl-D-galactosamine specific lectin from the fruiting bodies [33] and a D-rhaman specific lectin from the mycelium [44].

Solubility of proteins is greatly affected by presence of ions. As the ionic strength increases, the solubility of proteins decreases [45]. Ammonium sulphate has been used by many researchers for the salting out process due to its relatively low cost, availability of the pure material as well as its high solubility [32]. Proteins that resulted in the 0–30% fraction are those that easily aggregate, those that were in the 60–90% are those that are more soluble [46]. There was a greater lectin activity in the 30–60% fraction as shown in Fig 3, indicating that the *B*. *edulis* lectin was mostly in this fraction.

The results obtained in this study presents a new array for exploring the occurrence of lectins within Zimbabwe local mushrooms. Despite the report of lectin activity in mushrooms from several researchers, this is the first report on the isolation and partial purification of a mucin specific lectin from *Boletus edulis* found in Miombo woodlands of Zimbabwe. *Boletus* lectins have been found to have mitogenic activity towards tumour cells [16–18], antimicrobial activity as well as inducing interleukin-1 and interleukin-2 cytokines [19]. Mushroom lectins specific to mucin have antimicrobial activity [41] and antiproliferative activity as well as enhancing the immune system [38]. These previous findings spotlight great potential biological activities of the *B*. *edulis* lectin isolated in this study. Thus, research on the mucin specific lectin isolated in this study, may lead to information which could be used for the development of dietary supplements and other therapeutic agents. In addition, lectins with unique and distinctive glycan-binding selectivity can be exploited for probes of glycan structures and used in disease diagnostics.

## Conclusions

The present study demonstrates for the first time, the screening of wild mushrooms from Miombo woodlands of Zimbabwe for the occurrence of lectins and identifies a novel lectin from *B*. *edulis*. From the different mushrooms studied, *Amanita* species, *Boletus edulis* and *Lactarius kabansus* show varying levels of lectin activity with B. edulis having the highest lectin activity. The novel lectin from *B*. *edulis* is specifically inhibited by a complex glycoprotein, porcine mucin. Simple sugars cannot inhibit the haemagglutination activity of *B*. *edulis* lectin. The newly identified B. edulis lectin has potent biological activities as shown by other porcine specific lectins from previous research. The *B*. *edulis* lectin from this study is unique because it is from a locally available edible mushroom which can be easily incorporated as a supplement in diet unlike other lectins from non-edible mushrooms. This research highlights the importance of local wild mushrooms as valuable sources of novel lectins with unique specificity. The novel *B*. *edulis* lectin from this study needs further characterisation to determine its potent biological activities.

## Supporting information

S1 Table Composition of reagents used in haemagluttination assays. (PDF)

S2 Table Ammonium sulfate precipitation showing the weight (g) of ammonium sulfate added to one litre of solution to produce a desired change in the concentration (% saturation) of ammonium sulfate. (PDF)

S1 Fig Haemagglutination assay of three of the ten mushroom species with sheep erythrocytes showing representative results. (PDF)

S2 Fig Haemagglutination inhibition assay of B. edulis lectin by various sugars which had their concentration increased from 0.2 M to 0.5 M. There was no inhibition of B. edulis haemagglutination activity.
(PDF)
