# Supplementary material for: Screening of mushrooms from the woodlands of Zimbabwe: Occurrence of lectins and partial purification of a mucin specific lectin from Boletus edulis
Source: PLoS One. 2022 Apr 14;17(4):e0265494. doi: 10.1371/journal.pone.0265494 (PMC9009683; doi:10.1371/journal.pone.0265494)
Supplement: S1 Table — (PDF) [file pone.0265494.s001.pdf]

S1 Table. Composition of reagents used in haemagluttination assays.

Reagent	Composition
**0.9% Saline solution**	8.766 g NaCl, 2 g NaN_3, 0.9895 g $MnCl_2$ and 0.735 $CaCl_2$<br>Fill to 1 L with distilled water
**Alsever solution**	0.05 g glucose, 0.80 g Sodium citrate and 0.42 g NaCl<br>Fill to100 ml with distilled water
**0.1 M $NaHCO_3$ buffer pH 8.3 containing 0.5 M NaCl**	8.4 g of $NaHCO_3$ and 29.22 g of NaCl<br>Fill to 1 L with distilled water. Adjust to pH 8.3 using concentrated NaOH or HCl
**0.1 M Tris-HCl buffer pH 8 containing 0.5 M NaCl**	12.114 g Trizma and 29.22 g NaCl<br>Fill to 1 L with distilled water. Adjust to pH 8 using concentrated HCl
**0.1 M Tris-HCL buffer pH 8 containing 1 mM $CaCl_2$ and 1 mM NaCl**	0.15 g $CaCl_2$, 12.114 g Trizma and 0.058 g NaCl<br>Fill to 1 L with distilled water. Adjust to pH 8 using concentrated HCl
