# Supplementary material for: Screening of mushrooms from the woodlands of Zimbabwe: Occurrence of lectins and partial purification of a mucin specific lectin from Boletus edulis
Source: PLoS One. 2022 Apr 14;17(4):e0265494. doi: 10.1371/journal.pone.0265494 (PMC9009683; doi:10.1371/journal.pone.0265494)
Supplement: S2 Table — (PDF) [file pone.0265494.s002.pdf]

S2 Table. Ammonium sulfate precipitation showing the weight (g) of ammonium sulfate added to one litre of solution to produce a desired change in the concentration (% saturation) of ammonium sulfate.

**%**	**10**	**15**	**20**	**25**	**30**	**33**	**35**	**40**	**45**	**50**	**55**	**60**	**65**	**70**	**75**	**80**	**85**	**90**	**95**	**100**
**10**	56	84	114	144	176	196	209	243	277	313	351	390	430	472	516	561	610	662	713	767
**15**		28	57	86	118	137	150	183	216	251	288	326	365	406	449	494	540	592	640	694
**20**			28	57	88	107	120	153	185	220	256	294	333	373	415	459	506	556	605	657
**25**				29	59	78	91	123	155	189	225	262	300	340	382	424	471	520	569	619
**30**					30	49	61	93	125	158	193	230	267	307	348	390	436	485	533	583
**33**						19	30	62	94	127	162	198	235	273	314	356	401	449	496	546
**35**							12	43	74	107	142	177	214	252	292	333	378	426	472	522
**40**								31	63	94	129	164	200	238	278	319	364	411	457	506
**45**									31	63	97	132	168	205	245	285	328	375	420	469
**50**										32	65	99	134	171	210	250	293	339	383	431
**55**											33	66	101	137	176	214	256	302	345	392
**60**												33	67	103	141	179	220	264	307	353
**65**													34	69	105	143	183	227	269	314
**70**														34	70	107	147	190	232	275
**75**															35	72	110	153	194	237
**80**																36	74	115	155	198
**85**																	38	77	117	157
**90**																		39	77	118
**95**																			38	77
**100**																				39
