# Supplementary material for: Screening of mushrooms from the woodlands of Zimbabwe: Occurrence of lectins and partial purification of a mucin specific lectin from Boletus edulis
Source: PLoS One. 2022 Apr 14;17(4):e0265494. doi: 10.1371/journal.pone.0265494 (PMC9009683; doi:10.1371/journal.pone.0265494)
Supplement: S1 Fig — (PDF) [file pone.0265494.s003.pdf]

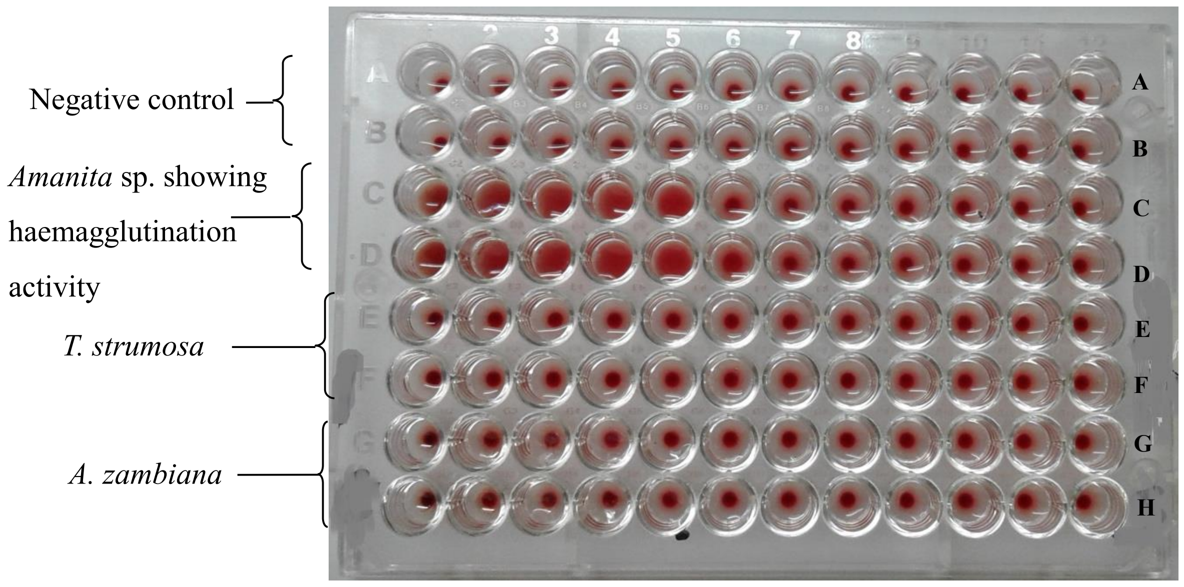

S1 Fig. Haemagglutination assay of three of the ten mushroom species with sheep erythrocytes showing representative results.
