# Supplementary material for: Screening of mushrooms from the woodlands of Zimbabwe: Occurrence of lectins and partial purification of a mucin specific lectin from Boletus edulis
Source: PLoS One. 2022 Apr 14;17(4):e0265494. doi: 10.1371/journal.pone.0265494 (PMC9009683; doi:10.1371/journal.pone.0265494)
Supplement: S2 Fig — There was no inhibition of B. edulis haemagglutination activity. (PDF) [file pone.0265494.s004.pdf]

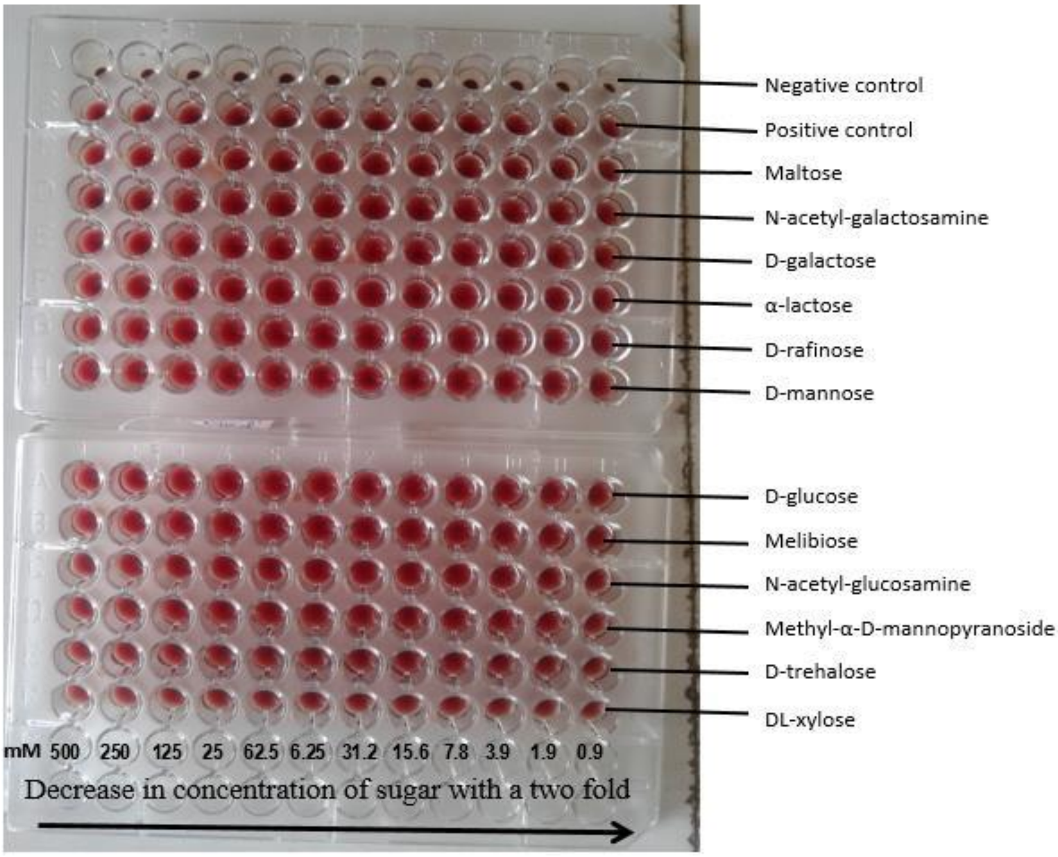

S2 Fig. Haemagglutination inhibition assay of *B. edulis* lectin by various sugars which had their concentration increased from 0.2 M to 0.5 M. There was no inhibition of *B. edulis* haemagglutination activity.
